# Mediators of Inflammation in Polycystic Ovary Syndrome in Relation to Adiposity

**DOI:** 10.1155/2010/758656

**Published:** 2010-04-08

**Authors:** Thozhukat Sathyapalan, Stephen L. Atkin

**Affiliations:** Academic Endocrinology, Diabetes and Metabolism, Hull York Medical School, Michael White Diabetes Centre, 220-236 Analby Road, Hull Royal Infirmary, Hull HU3 2JZ, UK

## Abstract

Polycystic ovary syndrome (PCOS) is a common endocrine disorder in women of reproductive age group and is associated with a higher cardiovascular risk. Obesity, mainly visceral adiposity, is prevalent in patients with PCOS. Obesity is associated with low-grade inflammation and raised inflammatory cytokines, both of which are also described in patients with PCOS. In this paper, the potential relationships between fat distribution, adipocyte dysfunction and, altered inflammatory markers in patients with PCOS have been discussed.

## 1. Obesity in Polycystic Ovary Syndrome (PCOS)

PCOS is one of the most common endocrine disorders in women of reproductive age with a prevalence of 5%–7% [1–3].PCOS is associated with a broad range of adverse sequelae, including hypertension, dyslipidemia, insulin resistance, hyperandrogenaemia, gestational and type 2 diabetes, which ultimately increase the risk of cardiovascular morbidity [4–12]. Obesity is present in varying degrees in women with PCOS and is associated with hyperandrogenaemia and insulin resistance [13].

## 2. Inflammatory Mediators in Obesity

Obesity is associated with a state of chronic systemic inflammation manifested by increased serum levels of inflammatory cytokines as well as alterations in peripheral blood lymphocyte frequencies and function [14–16]. These changes are present not only at the tissue level but also in adipose, liver, and other tissue beds [17, 18]. This inflammatory process could be the underlying cause of obesity-related comorbidities, including atherosclerosis, diabetes and steatohepatosis [19–23].

Obesity-related inflammation is often considered a disorder of innate immunity. However, there is a significant crosstalk between innate and adaptive immune systems, and indeed disorders of both innate and adaptive immunity have been implicated in obesity-related inflammation [17, 24, 25]. 

Inflammation is not only an acute response to trauma or infection, it is also a response to the ongoing processes of cell turnover associated with aging [26]. In this regard, the inflammatory response regulates fundamental processes intrinsic to cellular homeostasis, including proliferation, necrosis, and apoptosis. In keeping with the task of regulating tissue turnover, inflammatory responses are triggered not only by exogenous stimuli, but also by endogenous stimuli, such as the by-products of cell necrosis and apoptosis. For example, free fatty acids, systemic levels of which are elevated in obesity, are primary ligands for Toll-like receptors, central regulators of the innate immune response [27, 28]. Free fatty acids and Toll-like receptors therefore act as a direct link between the systems that regulate obesity and inflammation. 

At the molecular level, the intracellular signalling pathways that govern inflammation and glucose homeostasis demonstrate significant crosstalk and share multiple signalling mediators. At the cellular level, adipocytes and macrophages are closely related and likely evolved from a common primordial precursor cell [29], further evidence of the parallel evolution of inflammation and metabolic systems.

## 3. Inflammation and Insulin Resistance in Polycystic Ovary Syndrome

Approximately 60%–70% of PCOS patients are obese [30], with a central body fat distribution pattern described as visceral obesity that is well known to be highly associated with insulin resistance. However, PCOS patients have evidence of insulin resistance independent of obesity [31–33]. Insulin sensitivity is decreased by 35%–40% in women with PCOS, independent of obesity, a decrease similar in magnitude to that seen in T2DM mellitus [34]; however, any degree of obesity further impairs insulin action. About 50%–70% of all women with PCOS have some degree of insulin resistance [35]. It is now evident that PCOS has major metabolic consequences related to insulin resistance. Insulin resistance is associated with an increased risk for several disorders, including type 2 diabetes, hypertension, dyslipidemia (low high-density lipoprotein cholesterol and high triglycerides), elevated plasminogen activator inhibitor type 1 (PAI-1), elevated endothelin-1, endothelial dysfunction, and heart disease. 

Data have demonstrated a correlative as well as causative relationship between insulin resistance and inflammation [36]. Subclinical inflammation and insulin resistance are important predictors of cardiovascular disease [37]. Furthermore, in light of the role of insulin resistance in PCOS and of the increased cardiovascular risk of affected women, a relationship between inflammation and hormonal-metabolic features of women with PCOS has been demonstrated [38]. 

According to Rotterdam consensus criteria commonly used in clinical practice, two of the following three must be fulfilled for the diagnosis of PCOS: polycystic ovaries (12 or more follicles in each ovary, each follicle measuring 2–9 mm in diameter and/or ovarian volume >10 mL, one polycystic ovary is sufficient for the diagnosis), oligo-/anovulation; clinically diagnosed as oligo-/amenorrhea (menstrual cycles longer than 35 days or less than 10 menstruations per year) and hyperandrogenism (clinical or biochemical) [39]. In this consensus insulin resistance, metabolic syndrome, and obesity are not included in the diagnostic criteria to identify PCOS. However it is possible that some phenotypes of PCOS (i.e., those characterised by polycystic ovaries and oligo-menorrhea as per Rotterdam consensus criteria) may simply reflect abnormal androgen and/or LH production without having metabolic implications.

It has been reported that women with PCOS have significantly increased hs-C-reactive protein (hs-CRP) concentrations [40], suggesting CRP a marker of low-grade inflammation, as a predictor of coronary heart disease and cardiovascular events in PCOS that is also independently related to insulin resistance. The leukocyte count was found to be significantly higher in women with PCOS compared with healthy women, although no case of leukocytosis was found in either group [38]. Regarding the leukocyte differential, significant increases in lymphocytes and monocytes were observed in women with PCOS compared with controls, which might have been expected considering that they play a key role in the pathophysiological mechanism of atherosclerosis [38]. Inflammation has been recognised to play a central role in both initiation and progression of the atherosclerotic process; therefore, an elevated leukocyte count should be directly associated with increased incidence of coronary heart disease, ischemic stroke, and mortality from cardiovascular disease [41]. 

In patients with PCOS circulating levels of tumour necrosis factor *α* (TNF *α*), interleukin (IL)-6, hs-CRP, as well as white blood count (WBC) and neutrophil count have been found to be elevated compared with age- and /body mass index- (BMI-) matched controls [40, 42, 43]. In contrast, it has been shown that obesity, and not PCOS status per se, was a major determinants of the circulating inflammatory markers TNF *α*, soluble type 2 TNF receptor, IL-6, and hs-CRP [44, 45]. Increase in both low-grade chronic inflammation and insulin resistance in women with PCOS is associated with increased central fat excess rather than PCOS status [46]. Furthermore, TNF *α* is over expressed in adipose tissue [47] and induces insulin resistance through acute and chronic effects on insulin-sensitive tissues. The source of excess circulating TNF *α* in PCOS is likely to be adipose tissue in the obese but remains unknown in lean women with the disorder. However, increased visceral obesity could be a source of excess TNF *α* in lean women with PCOS.

Another proinflammatory cytokine is IL-18, which was reported to be increased in PCOS [48]. IL-18 induces the production of TNF *α* which promotes the synthesis of IL-6, which is also considered a strong risk marker for cardiovascular disease [49]. Collectively, the above findings indicate that low-grade chronic inflammation could be a novel mechanism contributing to increased risk of coronary heart disease in PCOS. 

Abdominal obesity is largely prevalent in obese women with PCOS [50]. Because of this, it is not surprising that the same alterations of abdominal obesity have been found in obese women with PCOS. In fact, compared with normal weight controls, obese women with PCOS present lower levels of adiponectin [51], increased levels of PAI-1 [52], increased activity of the angiotensin-renin system [53, 54], and increased cytokines and inflammatory markers [41]. However, obese patients with PCOS have more severe insulin resistance and higher androgen levels in comparison with non-PCOS women with abdominal obesity. Since both of these factors may affect adipocyte function, it is important to understand whether there are differences in production of adipose factors between obese women with PCOS and non-PCOS women with abdominal obesity. There were no differences in levels of leptin, resistin, and adiponectin between obese women with PCOS and obese controls [55]. There were also no differences in levels of TNF *α*, IL-6, and markers of inflammation between obese women with PCOS and obese controls [41, 44]. A significant increase in PAI-1 levels between obese women with PCOS and obese controls has been reported [56]. However, comparing normal-weight patients with PCOS with controls of similar BMI, normoweight women with PCOS have higher serum levels of PAI-1, TNF *α* and lower adiponectin than normoweight controls [41, 53, 55]. All these data suggest that normoweight women with PCOS have an increased production of adipokines that is similar to that found in abdominal obesity. Since these patients present a mild hyperinsulinemia and insulin resistance [57], it is possible that it is sufficient to alter the adipocyte function. Consistent with this hypothesis, serum PAI-1 correlates with serum insulin in normoweight women with PCOS [58, 59]. However, in the same group of patients, no correlation was found between serum adiponectin [55] or serum TNF *α* [41] and serum insulin levels or indices of insulin resistance. Even in overweight patients with PCOS no correlation between serum adiponectin and serum insulin or indices of insulin resistance was found [55].

Abundant leptin receptors have been detected in ovarian granulosa and theca cells [60]; furthermore, leptin treatment of these cells in vitro caused significant reduction in their steroid output [61]. It is possible that leptin has a dual effect on reproduction and that the major site of action differs according to the circulating levels [62]. Initial reports suggested that a substantial proportion of women with PCOS have leptin levels that are higher than expected for their BMI [63]. However subsequent studies have provided evidence that circulating leptin levels are fully accounted for by the degree of adiposity and BMI compared to matching control subjects [64–67]. On the other hand, it has also been reported that, for any given body weight, circulating leptin concentrations are lower in women with PCOS than those without, suggesting that neuroendocrine recognition of obesity may be impaired in such women [68].

Hyperinsulinemia alone is likely not sufficient to explain adipocyte dysfunction of normoweight women with PCOS. Theoretically, normoweight and overweight women with PCOS may have some degree of visceral obesity that is insufficient to effect an increase in body weight per se, but that may be sufficient to determine increased production of some adipokines [55]. On the other hand, visceral obesity and hyperinsulinemia are generally strictly related, and it is difficult to separate the two phenomena.

It has been shown that normoweight patients with PCOS have higher fat accumulation in visceral deposit [69] and lower subcutaneous fat in gluteofemoral area [70]. Although greater experiences and studies on the correlation between fat distribution and adipose products in normo-weight and overweight women with PCOS are needed, the available data suggest that in these patients, increased abdominal fat participates in the increased cardiovascular risk. Of course, insulin resistance is linked to the increase of visceral fat, and it may contribute to the adipocyte dysfunction of normoweight women with PCOS.

In conclusion, patients with PCOS present excessive fat accumulation in visceral deposits, and it plays an important role in their increased cardiovascular disease. This altered fat distribution is present not only in the obese, but also in normoweight patients with PCOS. Altered fat distribution and adipocyte dysfunction along with chronic lowgrade inflammation could be a novel mechanism contributing to increase in cardiovascular risk in PCOS.
